# Mobile Health (mHealth) Approaches and Lessons for Increased Performance and Retention of Community Health Workers in Low- and Middle-Income Countries: A Review

**DOI:** 10.2196/jmir.2130

**Published:** 2013-01-25

**Authors:** Karin Källander, James K Tibenderana, Onome J Akpogheneta, Daniel L Strachan, Zelee Hill, Augustinus H A ten Asbroek, Lesong Conteh, Betty R Kirkwood, Sylvia R Meek

**Affiliations:** ^1^Malaria Consortium AfricaKampalaUganda; ^2^Malaria ConsortiumLondonUnited Kingdom; ^3^Institute of Global HealthUniversity College LondonLondonUnited Kingdom; ^4^Faculty of Epidemiology and Population HealthDepartment of Population HealthLondon School of Hygiene and Tropical MedicineLondonUnited Kingdom

**Keywords:** mHealth, community health worker, Africa

## Abstract

**Background:**

Mobile health (mHealth) describes the use of portable electronic devices with software applications to provide health services and manage patient information. With approximately 5 billion mobile phone users globally, opportunities for mobile technologies to play a formal role in health services, particularly in low- and middle-income countries, are increasingly being recognized. mHealth can also support the performance of health care workers by the dissemination of clinical updates, learning materials, and reminders, particularly in underserved rural locations in low- and middle-income countries where community health workers deliver integrated community case management to children sick with diarrhea, pneumonia, and malaria.

**Objective:**

Our aim was to conduct a thematic review of how mHealth projects have approached the intersection of cellular technology and public health in low- and middle-income countries and identify the promising practices and experiences learned, as well as novel and innovative approaches of how mHealth can support community health workers.

**Methods:**

In this review, 6 themes of mHealth initiatives were examined using information from peer-reviewed journals, websites, and key reports. Primary mHealth technologies reviewed included mobile phones, personal digital assistants (PDAs) and smartphones, patient monitoring devices, and mobile telemedicine devices. We examined how these tools could be used for education and awareness, data access, and for strengthening health information systems. We also considered how mHealth may support patient monitoring, clinical decision making, and tracking of drugs and supplies. Lessons from mHealth trials and studies were summarized, focusing on low- and middle-income countries and community health workers.

**Results:**

The review revealed that there are very few formal outcome evaluations of mHealth in low-income countries. Although there is vast documentation of project process evaluations, there are few studies demonstrating an impact on clinical outcomes. There is also a lack of mHealth applications and services operating at scale in low- and middle-income countries. The most commonly documented use of mHealth was 1-way text-message and phone reminders to encourage follow-up appointments, healthy behaviors, and data gathering. Innovative mHealth applications for community health workers include the use of mobile phones as job aides, clinical decision support tools, and for data submission and instant feedback on performance.

**Conclusions:**

With partnerships forming between governments, technologists, non-governmental organizations, academia, and industry, there is great potential to improve health services delivery by using mHealth in low- and middle-income countries. As with many other health improvement projects, a key challenge is moving mHealth approaches from pilot projects to national scalable programs while properly engaging health workers and communities in the process. By harnessing the increasing presence of mobile phones among diverse populations, there is promising evidence to suggest that mHealth can be used to deliver increased and enhanced health care services to individuals and communities, while helping to strengthen health systems.

## Introduction

Community health workers were a cornerstone of primary health care as envisaged by the Declaration of Alma-Ata in 1978, yet the enthusiasm for community health workers started to diminish by the early 1990s, partly because of the challenges of scaling-up programs in a sustainable fashion while maintaining their effectiveness [1]. However, due to slow progress toward the United Nations’ Millennium Development Goals, community-based programs delivering care to sick children have yet again become priorities to curb child mortality in high-mortality countries [2]. Integrated community case management (ICCM) of malaria, pneumonia, and diarrhea delivered by community health workers is such a strategy, which is now being implemented at scale in several African countries, including Uganda and Mozambique. The purpose of ICCM is to improve access to effective treatment for sick children among hard-to-reach populations with the ultimate goal of reducing under-5 mortality.

The Innovations at Scale for Community Access and Lasting Effects (inSCALE) project, a collaboration between Malaria Consortium, London School of Hygiene and Tropical Medicine, and University College of London, aims to better understand community health workers motivation and supervision, and to find feasible and acceptable solutions to community health workers’ retention and performance, both of which are vital for successful implementation of ICCM at scale. One such solution could be to use mobile phones as a tool to increase the status of community health workers in the community and allow frequent feedback and support to community health workers based on data submitted, potentially resulting in improved quality of care delivered.

With almost 5 billion mobile phone users in the world, health care providers and researchers are realizing the potential of using mobile technologies, such as mobile phones, portable computers, and personal digital assistants (PDAs), for health services. Mobile health (mHealth), as defined by the World Health Organization (WHO), is an area of electronic health (eHealth) that provides health services and information via mobile technologies such as mobile phones and PDAs. mHealth can also support the performance of health care workers by the dissemination of clinical updates, learning materials, and reminders [3], particularly in underserved rural locations in low- and middle-income countries where community health workers deliver ICCM to children sick with diarrhea, pneumonia, and malaria [3,4].

The aim of this review is to provide a thematic overview of mHealth project approaches to the intersection of mobile technology and public health and the application of these approaches in programs specifically focusing on community health workers. The potential challenges and opportunities for integration of such mHealth applications in existing national systems are discussed.

## Methods

A 2-stage process was applied in this review. In the first stage, a broad search was done to generate a list of domains in which mHealth has been applied in low- and middle-income countries. Non-peer-reviewed sources of information, including Web-based mHealth portals, mHealth review documents [4-7], and reports that specifically map out mHealth initiatives in Uganda and Mozambique [8,9] were used (Table 1). The search in this stage did not aim to generate a comprehensive list of all mHealth projects conducted in low- and middle-income countries; the purpose was to generate different mHealth domains and give examples of projects that have addressed the domains identified.

**Table 1:** Sources used to identify mHealth projects.

Source	Name
mHealth portals	The Communication Initiative Network [10]
	KIT Royal Tropical Institute [11]
Review documents	Mechael et al [5]
	Vital Wave Consulting [6]
	Vital Wave Consulting [7]
Country reports	Macueve [8]
	Mwagale and Kakaire [9]

In the second review stage, a narrower and more systematic search was done to identify projects that applied mHealth for community health workers in low- and middle-income countries. In addition to the sources provided in Table 1, peer-reviewed papers were identified from searches on PubMed and Google Scholar by using the following search terms: mHealth, mobile health, developing, and low income. Projects conducted in countries with low, lower-middle, or upper-middle incomes (as per World Bank definition) qualified for inclusion if they mentioned that at least 1 of their user groups included community health workers. Although the main reviews for both stages 1 and 2 were conducted by the first author (KK) between June and September 2010, the review in stage 2 was updated in April 2012 by using the same search terms as used previously. Project descriptions published after these dates are not included in this review.

The information from stage 1 was structured by the first author (KK) who classified the projects into predefined themes. Various numbers of themes have been used previously within the mHealth ecosystem, ranging from 4 different categories in a review by Blynn [12], 5 in a paper by Mechael et al [5], and 6 in a report by Vital Wave Consulting [6]. To give a more diversified description of the projects reviewed, we adopted the 6-theme classification used by Vital Wave Consulting (Table 2).

**Table 2:** The six themes in mHealth.

Theme^a^	Description
Education and awareness	Primarily 1-way communication programs to mobile subscribers via SMS/text messaging in support of public health, behavior-change campaigns.
Data access	Applications designed to use mobile phones, PDAs, or laptops to enter and access patient data. Some projects may also be used by patients to access their own records.
Monitoring and compliance	One- or 2-way communications to the patient to monitor health conditions, maintain caregiver appointments, or ensure strict medication regimen adherence. Some applications may also include inpatient and outpatient monitoring sensors for monitoring of multiple conditions (such as diabetes, vital signs, or cardiac).
Disease and emergency tracking	Applications using mobile devices to send and receive data of disease incidence, outbreaks, geographic spread of public health emergencies, often in association with Global Positioning System (GPS) systems and backend applications for visualization.
Health information systems	Applications developed for “back office” or central health care information technology systems allowing for access by and integration with mHealth application. Such applications often tie-in to regional, national, or global systems.
Diagnosis and consultation	Applications developed to provide support for diagnostic and treatment activities of remote caregivers through Internet access to medical information databases or to medical staff.

^a^ Adopted from Vital Wave Consulting [6].

Key mHealth technologies reviewed included mobile phones, PDAs and smartphones, patient monitoring devices, and mobile telemedicine devices. The ways these tools can be used for education and awareness, data access, and for strengthening health information systems were explored. We also considered how mHealth may support patient monitoring, clinical decision making, and tracking of drugs, supplies, and emergencies.

For information gathered in stage 2 of the review process, the results were analyzed, classified into a theme, and described in more detail by using a predesigned data collection table (available on request from first author). The results were presented to the coauthors in an 8-person research team meeting and the classification was discussed until consensus was achieved.

## Results

### Common mHealth Solutions

The main capabilities provided by mHealth applications are voice, text, and data access with information going 1 way, 2 ways, or multiple ways (Table 3). These applications included phone calls (personal or automated robocalls with or without toll-free numbers), text messages (including personal text reminders or mass texting for community mobilization), data transfer for health record tracking or clinical decision support, and mobile telemedicine devices for patient monitoring or diagnosis.

**Table 3:** Example of mHealth applications utilizing 1-way, 2-way, or multiway communication.

Communication type	Example mHealth application
1-way	SMS/rapid SMS
	Sending data
	Push messages
	“Please call me” message
2-way	Sending data and receiving feedback
	Quizzes/games
	Hotlines/textlines
	Remote consultation/training
Multiway	Frontline SMS
	Facebook
	Twitter

### One-Way, Two-Way, or Multiway Applications

Communication between a sender and receiver can occur in more than 1 direction and within varying group sizes. One-way communication is similar to mass media that distributes information in 1 direction. mHealth innovations have typically been designed as 1-way communications in which projects use “push” technology to deliver information to subscribers’ phones by using messages tailored to personal needs. Most commonly identified push designs include bulk short message service (SMS) or robocalls to large audiences.

Two-way communication is interactive and more similar to interpersonal communication. For users, interactivity may require greater effort and generate greater interest. Interactive quizzes, information menus, data collection and tailored responses, hotlines, and interactive voice responses are examples of 2-way communication [5]. Although most 2-way communication does not occur in real time, some applications, such as closed user groups or voice over IP (VoIP) for remote health consultations and health worker training, do use real-time communication [6].

Multiway communication can vary the number of senders and receivers, including 1-to-many, many-to-1, and many-to-many communication. Many-to-many communications include social media applications, such as Facebook or Twitter, that can be accessed from most Internet-enabled mobile phones. Most mHealth projects used a combination of 1-way and 2-way communication methods pertinent to several themed categories in Table 2, whereas only a few projects could be identified that used social media.

### Education and Awareness

The Cellphones4HIV project in South Africa described by de Tolly and Alexander [13] sends out messages on antiretroviral treatment adherence using Unstructured Supplementary Service Data (USSD) (ie, the system used to load airtime), Mxit (a Java application that allows general packet radio service [GPRS] or 3G-based instant messaging) and voicemail messages pushed into the user’s voicemail inbox with notification by SMS. Push designs were found to have differing capabilities, limitations, and requirements, but may be combined, adapted, or further expanded as technology evolves.

Projects for remote health information dissemination, like Project Masiluleke [14] and Text-to-Change (TTC) [15], have reached large audiences with information on HIV prevention and treatment using ”please call me” (PCM) messages and bulk SMS. PCM messages have been widely used in mHealth projects in Africa because they are free for senders and can be sent from phones that have no credit. Project Masiluleke in South Africa sent 1 million PCM messages per day for 1 year, offering contact information for local HIV and tuberculosis call centers [14].Within 5 months, calls to South Africa’s National AIDS helpline quadrupled [16]. In Uganda, TCC used a bulk SMS platform to create dialog and increase awareness of HIV in order to reduce related stigma and discrimination, and motivate people to seek HIV testing and treatment [15]. TTC also sent out quizzes and information about HIV prevention and testing, awarding those who pass the quiz with airtime. Of 15,000 subscribers contacted by TTC, 2500 responded to each question.

In FHI360-SATELLIFE’s Uganda Health Information Network (UHIN) project, continuing medical education targeted to health workers was broadcast 3 times per week via PDAs regarding diagnosis, treatment, and prevention of major health problems [17]. In addition, they received daily news from mainstream media. Other projects used SMS for behavior-change communication. The Text2Teach project gave Philippine teachers a mobile phone texting platform to receive videos via satellite over school-based televisions and mobile technology involving parents [18]. Behavior-change communication can be used in various applications, from family planning and teenage pregnancy to disease awareness and prevention to advice on agricultural and farming techniques.

Social networks, such as Facebook, Twitter, or Hi5, are used by hundreds of millions of people to communicate about a huge range of topics, including health. The WHO used Twitter during the influenza A (H1N1) pandemic and, at time of writing, had more than 11,700 followers from all over the world [19]. In Mozambique, the nonprofit organization DKT International launched a social franchising program, branded as Intimo, that uses social media to increase access to its clinics. Its Facebook page reaches over 6600 Mozambicans (85% between the ages of 18 and 34 years) with information on family planning and reproductive health [20]. In Indonesia, the Fiesta condom brand has used Facebook, Twitter, and YouTube to talk about safe sex and condom use [21].

#### Community Health Worker Program Innovations for Education and Awareness

Through SMS with community members and community health workers, mHealth has opportunities to communicate health messages directly and simultaneously [22]. The SMS campaigns for health education, promotion, and awareness typically used SMS to disseminate information and prevention messaging or direct patients to services. Mobile phones also present opportunities for community health workers to communicate directly with one another and provide peer support [4]. To provide additional support to community health workers during home visits, the Tanzanian Mobile Video for Community Health Workers project used the CommCare tool to provide health education videos played on mobile phones [23].

Stakeholders suggested expanding 1-way to 2-way communications, including introducing a referral alert process in which community health workers call health facilities before the patients’ arrival [4]. Establishing call-in services for each health facility could also allow community health workers to receive updated information on drug stocks, attendance records, and other relevant information. In addition, appointment confirmation texts for referred patients with time, date, and appointment location could be effective, as well as SMS alerts to community health workers about appointments attended by referred patients. Texts or SMS could also be used by health facility workers and community health workers to keep each other informed of recent developments and upcoming events, including SMS to community health workers on their birthday for motivation [4].

The concern that national privacy laws can hinder projects from accessing the target beneficiaries’ personal phone numbers was raised. One stakeholder mentioned a project in which a collection of mobile phone numbers for health workers to send push messages had to be stopped after concerns were raised about the assumption that all health providers had given their permission to allow projects to reach them on their telephones (J Tibenderana, personal communication, September 2010).

### Data Access

Innovations in mHealth can conceivably change how data are used in health programs, leading to faster, decentralized decision making and reallocation of resources due to faster data analysis [22]. Handheld computers, PDAs, or laptops for data collection and reporting can use 1- or 2-way communication systems. RapidSMS has established a 2-way flow of communication that empowers stakeholders with a dynamic tool for fast, efficient, and accurate data collection, analysis, and communication [24]. In addition, SMS-based data for health care workers can identify, diagnose, and track patients by using streamlined technology that is automatically updated in a central system.

Twelve Ugandan projects used mobile technology for data collection and reporting [9]. Most were designed as 1-way communication systems to improve data collection or management in surveys, routine care, and vaccine trials.

#### Community Health Worker Program Innovations for Data Access

Although there is little evidence of the effectiveness of community health workers collecting and self-reporting data from patient records, mobile phones have been suggested as a useful tool for rural health workers’ reporting of data as it is suggested it improves accuracy, reduces time and cost, and improves data quality [19]. A cost-effectiveness study showed that using PDAs for data collection delivered 24% savings per unit of spending over traditional manual data collection and transmission approaches [25]. However, use of PDAs in a Rwandan ICCM program exacerbated, rather than lessened, volunteer workload [4]; mobile phone-assisted data collection became onerous and was felt to have distanced community health workers from the human side of their role, turning them into “data collection robots.”

Blaschke et al [26] and the Millennium Villages Project [27] describe the use of ChildCount+ that uses mobile technologies for improving data use and reporting among community health workers in several African countries, including Malawi and Uganda. This platform, developed by the Millennium Villages Project, aimed to improve maternal and child survival by supporting delivery of community-based management of acute malnutrition, malaria, and diarrhea. Three months after initiation, 95% of 9561 children under 5 years in the Malawian cluster had been registered using mobile technology, and only approximately 10% of incoming messages to the system were rejected due to improper formatting [26]. The RapidSMS platform used led to significant reduction in data transmission delay compared to Malawi’s current paper-based system.

### Monitoring and Compliance

Text messaging via mobile phones has garnered increasing attention as a means of reminding patients of appointments in the United Kingdom, United States, Norway, and Sweden. This resulted in a lowering of nonattendance to scheduled appointments, yielding significant savings in health costs for facilities and practitioners [28]. In this case, the benefit is cost-related rather than health outcome-related.

In addition, SMS has also been used as a way of monitoring patients’ medication compliance. However, literature on treatment compliance has focused primarily on management of chronic diseases, such as diabetes, smoking cessation, and breast cancer, in high-income countries and few examples exist from low- and middle-income countries [5]. A South African trial showed tuberculosis patients with increased compliance rates, and a Thai study showed that 90% of tuberculosis patients receiving daily SMS medication reminders adhered to treatment [7,12]. A Kenyan efficacy study provided 428 HIV patients with mobile phones and randomized patients to receive daily, weekly, or no SMS reminders. Treatment adherence was improved for patients receiving weekly, but not daily, SMS and treatment interruptions were less likely [29]. Adding words of encouragement to an SMS did not prove more effective and confidentiality was a concern.

To improve medicine compliance and adherence to antiretroviral drugs in Uganda, a medical container called Wisepill was used to transmit a cellular signal whenever opened, send weekly SMS at preset times, and provide interactive voice response [30]. A similar project, SIMpill, monitored adherence to tuberculosis drugs in South Africa [31]. Few randomized controlled trials studying treatment compliance were found, and statistically significant results were limited by sample size; mixed results have been found in other studies [32]. A strong focus on feasibility and usability was evident, with little connection to health outcomes [5].

Other mHealth applications can be used to improve compliance to guidelines by health workers. A proof-of-concept randomized controlled Kenyan trial on adherence to malaria treatment guidelines used 10 carefully designed SMSs with drug delivery instructions and an unrelated motivational message to aid rural health facility workers [33]. Both immediate and 6-month analyses showed improved malaria case management. The trial is undergoing cost-effectiveness analysis and qualitative analysis to examine possible added burdens on health workers.

#### Community Health Worker Program Innovations for Monitoring Compliance

A randomized controlled trial delivered SMS to community-based peer health workers in rural Uganda supporting antiretroviral treatment for HIV patients [34]. No virological differences in patient outcome over 26 months were observed, but limited qualitative data showed improvements in patient care, logistics, and broad support from health workers and patients. Improvements in peer health worker morale and confidence were reported; peer health worker-patient relationships improved, shifting burdens from staff-patient relationships. As compared to voice calls, reservations about the lack of immediate response via SMS were noted, privacy concerns were raised, and phone maintenance and charging were also problematic.

### Disease and Emergency Tracking

Several countries have used mHealth innovations for not only disease tracking, but also for supply tracking. The Foundation for Innovative New Diagnostics (FIND) deployed RapidSMS in 2 districts in Uganda and worked with health centers to submit and map weekly epidemiological records, malaria case management, and malaria medicine stock reports [35]. The platform EpiSurveyor has also been widely used for emergency response and tracking supplies. It allows users to download, fill, and send forms to central databases for real-time analysis [36].

Mobile phones and Web-based technologies have also been used for early warning of disease outbreaks. The Acute Encephalitis Syndrome Surveillance Information System (AESSIMS) project in India aimed to improve immunization services for Japanese B encephalitis, diphtheria, hepatitis B, measles, pertussis, tetanus, and polio by tracking diseases in real time [37].

Reports have described mobile technology use during natural disasters, including the earthquakes in China in 2008 and Haiti in 2010 [38,39]. Mobile phones were primarily used for tracking population movements, infectious disease reporting, and coordinating search and rescue missions. Studies investigating mobile phone use for telemedicine during emergencies found them effective for relatively fast and accurate in-transit patient treatment, sending images for diagnosis, and using video capabilities.

#### Community Health Worker Program Innovations for Disease and Emergency Warning Systems

As part of Cambodia’s malaria elimination strategy, the National Center for Parasitology, Entomology and Malaria Control (CNM), with technical support from Malaria Consortium and WHO, village malaria workers are trained to send SMSs to report malaria cases in real time [40]. These SMS messages also support the paper reporting that feeds into the health information system from the health centers. The project had low start-up costs, estimated at US $100 for each village malaria worker, which includes a mobile phone, subscriber identity module (SIM) card, solar charger, and training. Because of the effective cooperation with the private sector, all SMS messaging is free resulting in essentially zero maintenance costs [41].

In areas where outbreaks of disease occur, community health workers could use mHealth to track medicine stocks (eg, FIND) and report observed cases with daily case statistics delivered using FrontlineSMS [40]. Community health workers can also minimize the impact of outbreaks by disseminating educational information about disease prevention and handling. In the Healthy Child Uganda project, community health workers used mobile phones to send emergency alerts and requisition supplies to support ICCM activities in treating pneumonia, diarrhea, and malaria [42].

### Health Information Systems

Health administration systems are used for epidemiological research, tracking of indicators for monitoring and evaluation, and financial and cost reporting for supply management [6]. Mozambique used PDAs to support collection of data from health records [43]. The stand-alone system, known as “módulo básico,” has now been implemented in all provinces and districts in the country [44].

Several African countries, including Mozambique and Uganda, have tested 2-way access to district health information by using mobile phone networks and low-cost PDAs for data dissemination, collection and reporting, and email exchange [17,45]. The Mozambique Health Information Network (MHIN) set up data transfer via PDAs using wireless access points and a server located at the Ministry of Health in Mozambique. District health offices received data from health centers and used the network to monitor drug stocks and guide orders. Up to 50% improvement in data quality was observed. The MHIN services are expanding to additional districts and cost-benefit analyses comparing MHIN- and paper-based approaches are planned [45].

The same team who worked on MHIN also set up UHIN in Uganda [17]. Health workers used PDAs to collect and upload data and emails via infrared, Bluetooth, or Wi-Fi at rural health facilities. The access point sent data and messages via mobile networks to the server, which routed them to the correct recipients and sent return messages with data and health information.

The public-private SMS for Life project in Tanzania used mobile phones and electronic mapping technology to generate and deliver weekly information to health centers on malaria medicines [46]. The project proved successful, and medicine stock-out rates were significantly reduced within 21 days.

Sustainability of countrywide mHealth programs relies on incorporation with the national health care program of the country, yet few African countries have developed national eHealth or mHealth policies, strategies, or guidelines [5]. Much of this is because of the limited knowledge of what works, how it works, and how much it costs. An exception is Ethiopia, where a national policy for eHealth is about to be launched [47].

#### Community Health Worker Program Innovations for Health Information Systems

Few studies have examined health information and administration systems that include community health workers. The ICT4MPOWER project is a 3-year proof-of-concept project in Uganda aiming to increase health system effectiveness and empower community health workers in rural areas by aiding referrals and patient follow-up, while ensuring transfer of skills and knowledge to health workers [48]. The Tanzanian CommCare project provided a community health mobile platform, enabling community health workers to provide more efficient care and to receive better supervision [49]. Such projects indicate the great potential to link community health workers with health administration systems by using mobile technologies that would add value to government health policy, providing integrated health data and a dynamic picture of national health care provision.

### Diagnosis and Consultation

Use of electronic technologies to provide support for diagnosis, consultation, and treatment activities conducted by remote caregivers is increasingly common. Mobile phones can be used as respiratory or pulse rate counters, gestational age date calculators, drug dose calculators, drip rate calculators, and drug reminder alarms when installed in mobile phones and linked to a sensor [50]. Another example of a diagnostic tool is CellScope, which uses a modified mobile phone for blood, urine, or other sample loading for malaria, HIV, and tuberculosis diagnosis [51]. None of these applications requires any transfer of data; hence, running costs are close to zero.

A pilot study of Electronic Integrated Management of Childhood Illnesses (eIMCI) in rural Tanzania, tested whether PDAs could improve diagnosis of children using IMCI protocols. The project was found to be feasible and acceptable to health workers in providing mobile decision support [52]. In addition, 6 Ugandan projects used mobile phones to send medical test results through SMS or email to patients and health workers; others used wireless devices to provide clinical training and patient care support services [9].

#### Community Health Worker Program Innovations for Diagnosis and Consultation

RapidSMS can be used in various ways, including supporting community health worker-patient interactions [24]. Mobile phones used as job aides could allow community health workers, via SMS or data transfer, to send patient information and receive instructions on how to proceed [27]. This could demonstrate program effectiveness to community health workers, potentially motivating continued work and better service [4]. In Colombia, the CellPhone GuideView system broke down complex diagnostic and treatment procedures into simple steps for community health workers using an authoring tool in which text, pictures, audio, and video were embedded to aid comprehension and ease of use [50,53]. Community health workers were then able to transmit images, data, and audio to remote experts for further advice.

## Discussion

The review revealed that there are very few formal outcome evaluations of mHealth in low-income countries. Although there is vast documentation of project process and uptake, most were evaluations of small-scale pilot studies that were not designed to demonstrate an impact on behavior change or health. There is also a lack of mHealth applications and services operating at scale in low- and middle-income countries. The most commonly documented use of mHealth was 1-way text-message and phone reminders to encourage follow-up appointments, healthy behaviors, and data gathering. Two-way communication applications focused primarily on data transmission with automated feedback response, and few projects were implementing real-time communication. Although some claim that social media can be an effective tool for engaging patients online [54], others argue that health institutions need to develop clear policies about the use of social media in patient care environments to ensure patient safety [55]. However, the majority of multiway and social media projects identified in this review were patient/user driven, such as Facebook or Twitter, with little or no involvement of treating physicians or nurses.

A limited number of mHealth projects were found which specifically targeted community health workers. Of the few projects identified, most used a combination of simple mobile phone applications for data submission, job aids to improve diagnostics, and for sending and receiving SMS messages and reminders. None of these projects had evaluated the impact of these tools on community health workers quality of care provided. Most projects used applications that communicated by using 1-way or 2-way SMS, whereas GPRS-enabled applications were rare. Although several projects tested applications that aimed to improve accuracy in community health worker data submission and clinical decision-making skills using electronic job aids [26,27,49], international stakeholders cautioned that these may result in community health workers focusing more on the technology than on the patient [4].

The key considerations for successful use of or expansion of mHealth innovations include collaboration, financing, literacy and cultural, partnerships, and technical considerations (Table 4). As a young field, mHealth is well positioned to benefit from best practices and available technology documented in various project reports. Sustainability and scalability are still the main challenges to the strategic deployment of mHealth applications, partly reflecting the gap between what application developers are doing on the ground and what the governments see as priorities and initiatives they need to step in and support [2]. Establishing true partnerships with users and policy makers throughout the design and implementation processes is critical for success and collaboration with operators could ensure technical support, make scale-up possible, and reduce costs to drive mHealth demand and innovation [4,5]. This is illustrated by examples from Ghana and Cambodia, where physicians registered with the Ghana Medical Association have access to unlimited calls through the mobile service operator, OneTouch [3], and where village malaria workers in Cambodia report malaria cases by using free SMS with Mobitel [40].

**Table 4:** Key considerations for successful use and expansion of mHealth innovations.

Areas of consideration	Description
Collaboration	Collaborative projects allow simpler widespread implementation
	Collaboration with operators could ensure technical support and make program scaling possible
	Collaboration more likely when all partners display strong affinity to the goal
Costs and sustainable financing	Collaborations can provide resources and support for project costs
	Organizations more likely to commit resources for piloting new initiatives when projects lasted for limited timeframe, when partners maintain control over deliverables, and when funds do not need approval and transference to third party
	Average SMS cost does not exceed US $0.05
	Lost phones and hardware can be mitigated by providing cheaper phones and ensuring equipment bears the program logo
Literacy and cultural specificity	Illiteracy is an important consideration for text-based innovations in low- and middle-income countries
	Accessibility to target users/patients must consider cultural sensitivities
Health worker partnership, engagement, training, and compliance	Partnership with users can enhance design and implementation of projects
	Using iterative cycles with target users when developing a software can stimulate ownership and enhance project engagement
	Data feedback reports should be distributed to users submitting data regularly (perhaps initially once per week)
	Use brief and personal SMS messages, allow opt out, allow language choice (with careful translation), and validate content with target users
Technical considerations	Use user-friendly and project-appropriate equipment
	Maintain 160 character length for SMS
	Take care with abbreviations, slang, and tone in SMS messages
	Ensure enough time for procuring and establishing necessary equipment and phone lines
	First resolve lack of telephone and Internet connectivity among target health care providers
	Use of existing technology, such as “please call me” messages, rather than introduction of new technologies
	Implement clear data usage and storage guidelines with data quality checks and backups made frequently

The national ownership of mHealth applications cannot be overemphasized. Some good examples of country ownership exist, such as state programs in Ghana and Nigeria, which address maternal and neonatal health using mobile phones [56]. The challenge is to have health ministers and officials at the same table as mobile service providers, doctors, technologists, and financiers. Coordination among these stakeholders and agreement of incentive structures and responsibilities for meaningful collaboration is needed to better inform public and private investments and the deployment of commercially viable solutions [5].

The mHealth interventions often used SMS to provide information, motivate individuals, and encourage self-management or promote disease prevention. However, illiteracy is an issue for text-based prevention interventions [5]. Culturally specific provision of health information is important because poorly designed campaigns can have negative unintended effects; good understanding of cultural context and strategies to overcome language and literacy barriers are needed. As with other mHealth applications, there is a significant gap in evidence on behavioral, social, economic, and health outcomes of using mobile phones and SMS for improving health in low- and middle-income countries, as demonstrated in a systematic review of the literature [57].

Funding in low- and middle-income countries is not adequate to support complex telemedicine in emergency situations. Infrastructural limitations, such as network capacity, also constrain the effectiveness of emergency monitoring and tracking [5]. However, routine data from all active SIM cards could be used in disaster-affected areas for near real-time monitoring of population movements during disease outbreaks [39]. Another significant barrier to implementation of mHealth systems is in relation to health worker resistance to new technology and broader discussion and research about health worker benefits, and incentives for use and compliance is required [5,6,22]. This would include ensuring adequate training also remains a critical component for large-scale implementation [5].

Addressing security and privacy issues in mHealth has also proven challenging. Guidelines on the rights to data, usage, and storage must be outlined and implemented, with sufficient qualitative data to explain potential findings collected alongside close program monitoring. For mHealth success, cooperation between local communities and regional and national health information systems is essential [3,58,59]. It is also unclear from the review whether SMS projects for health workers need to comply with any national privacy laws because collecting health workers’ private phone numbers to push messages is assuming that they have all given their permission to have the project reach them on their phones.

Limitations related to the landscape analysis should be considered when interpreting the results. The review focused only on 6 major thematic areas for mHealth and it is possible that some mHealth applications and tools have been excluded. Given the bulk of projects piloting mHealth applications in low- and middle-income countries, the first stage of the review only describes a sample of projects and applications tested under each thematic area. However, the second stage of the review, ie, that of mHealth projects targeting community health workers, was deemed systematic and comprehensive. The sources of the information reviewed were primarily obtained from project websites because few peer-reviewed evaluations were identified, potentially resulting in overreporting of positive results and underreporting of challenges or failures.

With partnerships forming between governments, technologists, non-governmental organizations, academia, and industry, there is great potential to improve health services delivery using mHealth in low- and middle-income countries. As with many other health improvement projects, a key challenge is moving mHealth approaches from pilot projects to national scalable programs while properly engaging health workers and communities in the process. By harnessing the increasing presence of mobile phones among diverse populations, there is promising evidence to suggest that mHealth can be used to deliver increased and enhanced health care services to individuals and communities, while helping to strengthen health systems.

## Abbreviations

AESSIMS: Acute Encephalitis Syndrome Surveillance Information System
FIND: Foundation for Innovative New Diagnostics
GPRS: general packet radio service
GPS: Global Positioning System
ICCM: integrated community case management
MHIN: Mozambique Health Information Network
PCM: please call me
PDA: personal digital assistant
SIM: subscriber identity module
SMS: short message service
TTC: Text-to-Change
UHIN: Uganda Health Information Network
USSD: Unstructured Supplementary Service Data
VoIP: voice over IP
WHO: World Health Organization

## References

[1] The World Bank (1993). World Development Report 1993: Investing in Health.

[2] United Nations Development Program (2012). Mobile technologies and empowerment: enhancing human development through participation and innovation.

[3] Mechael PN (2009). The case for mHealth in developing countries. Innovations: Technology, Governance, Globalization.

[4] Strachan DL, Källander K, Ten Asbroek AH, Kirkwood B, Meek SR, Benton L, Conteh L, Tibenderana J, Hill Z (2012). Interventions to improve motivation and retention of community health workers delivering integrated community case management (iCCM): stakeholder perceptions and priorities. Am J Trop Med Hyg.

[5] Mechael PN, Batavia H, Kaonga N, Searle S, Kwan A, Goldberger A, Fu L, Ossman J (2010). Barriers and Gaps Affecting mHealth in Low and Middle Income Countries: Policy White Paper.

[6] Vital Wave Consulting (2008). mHealth in the Global South Landscape Analysis.

[7] Vital Wave Consulting (2009). mHealth for Development: The Opportunity of Mobile Technology for Healthcare in the Developing World.

[8] Macueve G (2010). eHealth and mHealth in Mozambique: innovations likely to lead to increased retention and performance of Community Based Agents (CBAs).

[9] Mwagale S, Kakaire F (2010). Mobile Health Projects in Uganda.

[10] (2010). ICT for Development Network.

[11] (2010). Royal Tropical Institute: mHealth in Low-Resource Settings.

[12] Blynn E (2009). Piloting mHealth: A Research Scan.

[13] de Tolly K, Alexander H (2009). Soul Beat Africa-HIV/AIDS.

[14] (2010). Frog.

[15] aventh (2011). Soul Beat Africa.

[16] Tischler L (2009). Fast Company.

[17] (2010). FHI360-SATELLIFE Health Information and Technology.

[18] Trucano M (2009). EduTech.

[19] Anantraman V, Mikkelsen T, Khilnani R, Kumar VS, Pentland A, Ohno-Machado L (2002). Open source handheld-based EMR for paramedics working in rural areas. Proc AMIA Symp.

[20] (2010). SIMpill.

[21] Déglise C, Suggs LS, Odermatt P (2012). Short message service (SMS) applications for disease prevention in developing countries. J Med Internet Res.

[22] Waruingi M, Underdahl L (2009). AJFAND Online.

[23] aventh (2011). Soul Beat Africa.

[24] (2009). RapidSMS.

[25] Tumwesigye NM (2010). Uganda Health Information Network.

[26] Blaschke S, Bokenkamp K, Cosmaciuc R, Denby M, Hailu B, Short R (2009). MobileActive.org.

[27] Millennium Villages Project (2010). ChildCount+.

[28] (2006). The Economist.

[29] Pop-Eleches C, Thirumurthy H, Habyarimana JP, Zivin JG, Goldstein MP, de Walque D, MacKeen L, Haberer J, Kimaiyo S, Sidle J, Ngare D, Bangsberg DR (2011). Mobile phone technologies improve adherence to antiretroviral treatment in a resource-limited setting: a randomized controlled trial of text message reminders. AIDS.

[30] (2012). DKT International.

[31] McNab C (2009). What social media offers to health professionals and citizens. Bull World Health Organ.

[32] Derenzi B, Borriello G, Jackson J, Kumar VS, Parikh TS, Virk P, Lesh N (2011). Mobile phone tools for field-based health care workers in low-income countries. Mt Sinai J Med.

[33] Zurovac D, Sudoi RK, Akhwale WS, Ndiritu M, Hamer DH, Rowe AK, Snow RW (2011). The effect of mobile phone text-message reminders on Kenyan health workers' adherence to malaria treatment guidelines: a cluster randomised trial. The Lancet.

[34] Chang LW, Kagaayi J, Arem H, Nakigozi G, Ssempijja V, Serwadda D, Quinn TC, Gray RH, Bollinger RC, Reynolds SJ (2011). Impact of a mHealth intervention for peer health workers on AIDS care in rural Uganda: a mixed methods evaluation of a cluster-randomized trial. AIDS Behav.

[35] Asiimwe C, Gelvin D, Lee E, Ben Amor Y, Quinto E, Katureebe C, Sundaram L, Bell D, Berg M (2011). Use of an innovative, affordable, and open-source short message service-based tool to monitor malaria in remote areas of Uganda. Am J Trop Med Hyg.

[36] (2010). Datadyne.

[37] (2010). PATH.

[38] Yang C, Yang J, Luo X, Gong P (2009). Use of mobile phones in an emergency reporting system for infectious disease surveillance after the Sichuan earthquake in China. Bull World Health Organ.

[39] Bengtsson L, Lu X, Thorson A, Garfield R, von Schreeb J (2011). Improved response to disasters and outbreaks by tracking population movements with mobile phone network data: a post-earthquake geospatial study in Haiti. PLoS Med.

[40] Malaria Consortium, World Health Organization (2011). Moving Towards Malaria Elimination: Tools for Strengthening Malaria Surveillance in Cambodia.

[41] Nariddh MC (2011). Containment.

[42] (2010). Healthy Child Uganda.

[43] Macueve G (2009). e-Government Implementation in Mozambique: Transferring Lessons from the public sector. South African Computer Journal.

[44] Macanze J (2007). Monitoring and Evaluation Report of PDAs for Malaria Monitoring in Maputo Province, Mozambique - Final Report.

[45] (2010). FHI360-SATELLIFE Health Information and Technology.

[46] (2010). Roll Back Malaria.

[47] WHO Global Observatory for eHealth (2006). World Health Organization.

[48] (2010). SPIDER.

[49] (2010). Dimagi.

[50] Iyengar MS (2010). Microsoft Case Studies.

[51] (2010). CellScope.

[52] (2010). Dimagi.

[53] Iyengar MS (2009). slideshare.

[54] Bocock P (2010). Global Health Impact: Management Sciences for Health.

[55] Purdy C (2012). HealthWorks Collective.

[56] Sharp JW (2011). eHealth.

[57] World Health Organization, UNICEF (2012). Unicef.

[58] Braa J, Macome E, Mavimbe J, Nhampossa L, Cossa J, Jose B, Manave A, Sitói A (2001). The Electronic Journal of Information Systems in Developing Countries.

[59] Kimaro H, Nhampossa L (2005). Analyzing the problem of unsustainable health information systems in less?developed economies: case studies from Tanzania and Mozambique. Information Technology for Development.

